# The isopeptidase inhibitor 2cPE triggers proteotoxic stress and ATM activation in chronic lymphocytic leukemia cells

**DOI:** 10.18632/oncotarget.9742

**Published:** 2016-05-31

**Authors:** Andrea Tomasella, Raffaella Picco, Sonia Ciotti, Andrea Sgorbissa, Elisa Bianchi, Rossella Manfredini, Fabio Benedetti, Valentina Trimarco, Federica Frezzato, Livio Trentin, Gianpietro Semenzato, Domenico Delia, Claudio Brancolini

**Affiliations:** ^1^ Department of Medical and Biological Sciences, Università degli Studi di Udine, Udine, Italy; ^2^ Centre for Regenerative Medicine “Stefano Ferrari”, Department of Life Sciences, University of Modena and Reggio Emilia, Modena, Italy; ^3^ Dipartimento di Scienze Chimiche e Farmaceutiche, Università degli Studi di Trieste, Trieste, Italy; ^4^ Department of Medicine, Hematology and Clinical Immunology Branch, Padua University School of Medicine, Padua, Italy; ^5^ Department of Experimental Oncology and Molecular Medicine, Fondazione IRCCS Istituto Nazionale dei Tumori, Milan, Italy

**Keywords:** CLL, apoptosis, proteasome, proteotoxic stress, deubuquitylases

## Abstract

Relapse after treatment is a common and unresolved problem for patients suffering of the B-cell chronic lymphocytic leukemia (B-CLL). Here we investigated the ability of the isopeptidase inhibitor 2cPE to trigger apoptosis in leukemia cells in comparison with bortezomib, another inhibitor of the ubiquitin-proteasome system (UPS). Both inhibitors trigger apoptosis in CLL B cells and gene expression profiles studies denoted how a substantial part of genes up-regulated by these compounds are elements of adaptive responses, aimed to sustain cell survival. 2cPE treatment elicits the up-regulation of chaperones, proteasomal subunits and elements of the anti-oxidant response. Selective inhibition of these responses augments apoptosis in response to 2cPE treatment. We have also observed that the product of the ataxia telangiectasia mutated gene (ATM) is activated in 2cPE treated cells. Stimulation of ATM signaling is possibly dependent on the alteration of the redox homeostasis. Importantly ATM inhibition, mutations or down-modulation increase cell death in response to 2cPE. Overall this work suggests that 2cPE could offer new opportunities for the treatment of B-CLL.

## INTRODUCTION

B-cell chronic lymphocytic leukemia (B-CLL) is the most prevalent leukemia in Western countries and it is characterized by accumulation of malignant cells in the blood, lymph nodes, spleen and bone marrow. B-CLL is a severe disease with heterogeneous clinical course and although new therapies have significantly prolonged the overall survival, most patients relapse [1]. Augmented expression of anti-apoptotic Bcl-2 family members and up-regulation of pro-survival pathways contribute to the resistant phenotype [1–5].

Among drugs tested for the ability to trigger apoptosis in B-CLL cells, inhibitors of the ubiquitin-proteasome system (UPS) have raised some interest. Bortezomib, the first UPS inhibitor approved for the use in clinic, efficiently triggers apoptosis in *in vitro* cultured B-CLL cells [6, 7]. Unfortunately, clinical trials evaluating bortezomib in B-CLL patients were unsatisfactory [8]. Several constrains can explain this failure, including the chemical reaction between the boronate moiety of bortezomib and dietary flavonoids [9]. Furthermore bortezomib induces thrombocytopenia and neuropathy possibly due to proteasomal independent activities [10]. Hence, evaluating alternative compounds targeting the UPS for the treatment of B-CLL is of primary importance.

Small molecules characterized by the presence of a cross-conjugated α,β-unsaturated dienone with two sterically accessible electrophilic β-carbons can act as Michael acceptors to target nucleophiles, such as cysteine residues [11–14]. Highly susceptible to these compounds are the isopeptidases, which contain a cysteine in the catalytic core. Isopeptidases include DUBs (deubiquitylases) and ubiquitin-like proteases. Although the presence of different groups, in addition to the pharmacophore, can enhance or limit the promiscuity of these compounds, we refer to them as partially-selective isopeptidase inhibitors (P-SIIs) [11–16].

P-SIIs are potent inducers of apoptosis and of additional types of cell death, particularly in cells showing extreme apoptotic resistance [17–19]. We have recently developed a PEG-conjugated P-SII, named 2cPE optimized for the *in vivo* delivery. 2cPE is a pro-drug version of G5 [11], which can be activated by secreted esterase and exhibits promising anti-neoplastic activities *in vivo* [20]. In this manuscript, we have investigated the effect of 2cPE against B-CLL cells, in comparison with bortezomib. Our results prove that induction of proteotoxic stress is a key aspect of 2cPE activity and discovered an unexpected contribution of ATM in influencing 2cPE-induced apoptosis.

## RESULTS

### The UPS inhibitors bortezomib, G5 and 2cPE cause loss of viability of CD19^+^ B-CLL cells

**Figure 1:**
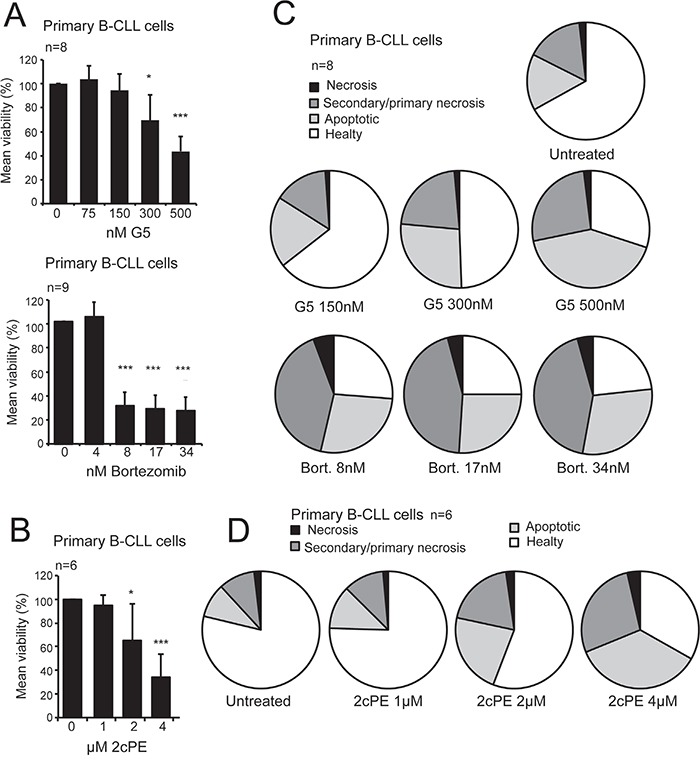
Pro-apoptotic activity of bortezomib, the P-SII G5 and its pro-drug derivative 2cPE in primary B-CLL cells **A.** Primary B-CLL cells viability following treatment with escalating doses of G5 or bortezomib for 24 hours as indicated. Cell viability was calculated as percentage of cells negative to PI and Annexin V staining after cytofluorimetric analysis. **B.** Flow cytometry analysis for apoptotic markers (Annexin V/PI) in order to define the type of cell death. Primary B-CLL cells were treated with the indicated concentrations of bortezomib or G5 for 24 hours. **C.** Primary B-CLL cells viability following treatment with escalating doses of 2cPE for 24 hours as indicated. Cell viability was calculated as the percentage of cells negative to PI and Annexin V staining after cytofluorimetric analysis. **D.** Flow cytometry analysis for apoptotic markers (Annexin V/PI) in order to define the type of cell death. Primary B-CLL cells were treated with the indicated concentrations of 2cPE for 24 hours. Columns, mean loss of viability + SD. *=p<0.05; **=p<0.01; ***p=<0.005.

Bortezomib and the isopeptidase inhibitor G5, or its PEGylated derivative 2cPE, induce loss of viability in primary CLL cells (Figure 1A and 1B). Cytofluorimetric analysis proved that, for all inhibitors, the loss of viability is largely caused by the induction of apoptosis, with only a minor fraction of the cells exhibiting markers (Annexin-V^−^ and PI^+^) of primary necrosis (Figure 1C and 1D).

### Gene expression profiles of B-CLL cells treated with the UPS inhibitors bortezomib and 2cPE

To explore whether bortezomib and 2cPE elicit similar or different biological responses, we performed microarray experiments in primary B-CLL cells. Leukemia CD19^+^ B-cells from 10 different patients were treated or not for 3, 6, 12 and 24 hours with 6nM of bortezomib or with 4μM of 2cPE. Under these conditions the two compounds induce equivalent levels of apoptosis, at 24 hours. For the microarray analysis the 6 hours time-point was selected in order to observe early adaptive responses to the inhibitors and to exclude changes in mRNA expression depending on cellular demise. The clinical and prognostic features of each of the 10 primary CLL samples and their responsiveness in terms of apoptosis are described in Table 1.

**Table 1:** Clinical characteristics, apoptotic response, mutational status and genetic alterations of the included patients

code	Sex/age	IGHV	FISH	RAI stage	CD38	2cPE	Bortezomib
LL300	F/79	U	13q-	II	NEG	88	89
LLC122	M/66	U	11q-	0	POS	15	59
LLC195	M/69	U	11q-; 13q-	II	POS	2	86
LLC351	M/67	U	17q-; 13q-	I	POS	81	92
LLC270	M/73	U	11q-	II	POS	17	65
LLC4	F/73	M	*	I	NEG	82	81
LLC305	M/56	M	Normal	0	NEG	75	54
LLC366	F/69	*	13q-; 12+; p53 mut	0	POS	45	77
LLC37	F/79	M	13q-	II	NEG	8	38
LLC43	M/83	M	12+	I	POS	68	82
						**Apoptosis at 24 hours (%)**	

Note: The gender of the patients, the IgHV mutational status (U=unmutated, M= mutated), CD38 expression (POS = positive for CD38 expression; NEG = negative CD38 expression), the cytogenetic alterations observed and the % of apoptosis after 24 of treatment with the indicated drugs for each patient sample are shown.

* indicates that the status of a specific characteristic for a patient is unknown.

**Figure 2:**
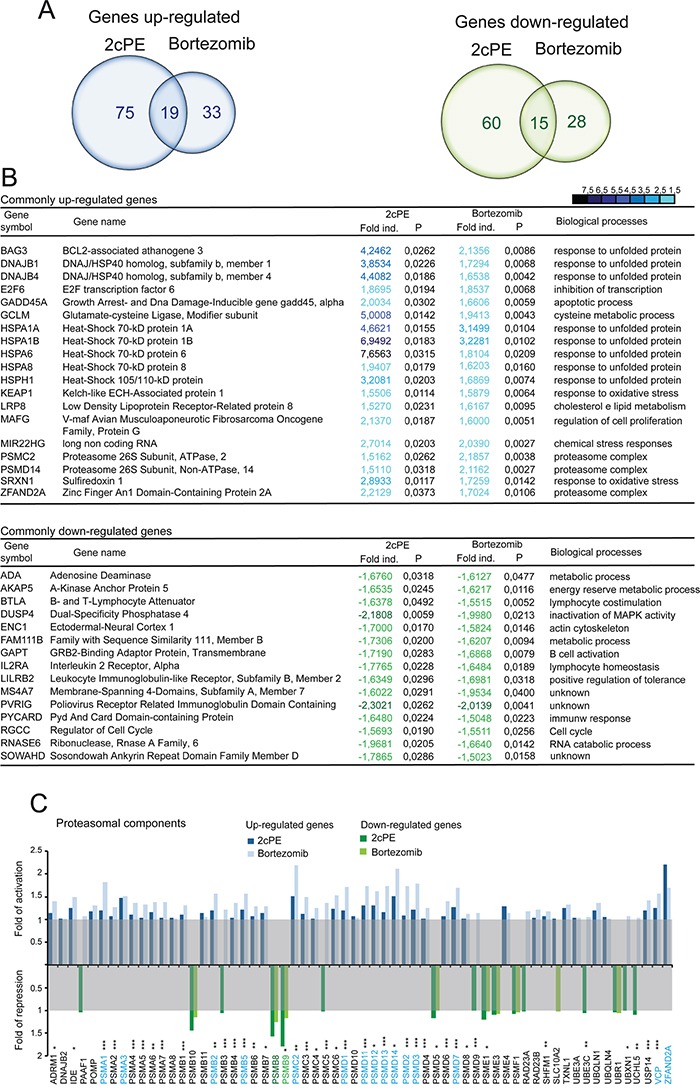
mRNA expression profiling of bortezomib and 2cPE treated B-CLL cells **A.** Venn diagram of commonly down (green) and up-regulated (blue) genes (fold changes >1.5; *P* values <0.01) using the paired t-test, in leukemic B-cell from 10 different patients after 2cPE and bortezomib treatments. **B.** List in alphabetic order of the commonly up- and down-regulated genes in leukemic B-cells form the different patients in response to 2cPE and bortezomib treatments (mRNA fold changes >1.5; <-1.5). In the heat map positive values are displayed in blue and negative in dark green. A colour code was selected to illustrate the fold changes. **C.** mRNA fold changes of the different proteasomal components after 2cPE and bortezomib treatments as indicated by the different colours.

Data analysis identified a list of common genes, which expression is influenced by both compounds. Venn diagrams (Figure 2A) illustrate that only few genes were commonly modulated by 2cPE and bortezomib in different patients (19 up-regulated and 15 down-regulated). Overall 2cPE influences the expression of a wider number of genes compared to bortezomib, 169 and 95 genes respectively, including both up and down-regulated ones.

The vast majority of the commonly up-regulated genes encode for chaperones implicated in the regulation of protein folding (Figure 2B). A second group of genes comprises proteasome subunits. Interestingly among the commonly down-regulated genes, metabolic elements and components of signaling pathways can be found.

It is known that the level of proteasomal subunits can influence responsiveness to bortezomib treatment [21–23]. Hence, we compared changes in the expression levels of all proteasomal genes. Up-regulation of mRNAs encoding for several proteasomal subunits is a mark of the response to both inhibitors. This response was more pronounced in the case of bortezomib, whereas the down-regulation of the immunoproteasome subunits PSMB8, PSMB9 and PSMB10 was comparable or even more evident in 2cPE treated cells.

The GO enrichment analysis for 2cPE and bortezomib regulated genes (Table 2) showed that the two drugs can elicit common but also distinct responses. Over-representations of processes such as protein folding, response to unfolded protein and response to organic substance characterize the genetic programs activated by 2cPE. Instead after bortezomib treatment, proteasome complex and regulation of E3 ligase activity are the most represented categories. Comparative analysis of the GO terms enrichment confirmed that the response to 2cPE treatment is strongly characterized by the up-regulation of chaperones, whereas the response to bortezomib is highly represented by UPS components (Supplementary Table S1).

**Table 2:** GO-Terms enrichment analysis

Gene enrichment analysis in 2cPE treated primary B-CLL cells						
GO-Term	Count	%	p Value	Benjamini	FDR	Fold Enr
GO:0006986~response to unfolded protein	13	8,33	1,66E-12	2,67E-09	2,78E-09	19,82
GO:0051789~response to protein stimulus	13	8,33	3,90E-07	1,96E-07	4,08E-07	13,15
GO:0050865~regulation of cell activation	14	8,97	7,16E-09	3,84E-06	1,20E-05	8,66
GO:0010033~response to organic substance	25	16,02	3,11E-08	1,25E-05	5,21E-05	3,75
GO:0006457~protein folding	13	8,33	7,73E-08	1,75E-05	0,00013	7,94
GO:0042981~regulation of apoptosis	25	16,02	2,38E-07	4,80E-05	0,0004	3,37
**Gene enrichment analysis in Bortezomib treated primary B-CLL cells**						
**GO-Term**	**Count**	**%**	**p Value**	**Benjamini**	**FDR**	**Fold Enr**
GO:0000502~proteasome complex	14	15,73	4,31E-18	5,26E-16	4,97E-15	42,52
GO:0051351~positive regulation of ligase activity	15	16,85	1,35E-18	1,16E-15	2,09E-15	37,56
GO:0043161~proteasomal protein catabolic process	15	16,85	1,91E-16	1,91E-14	3,44E-13	26,88

### Adaptive responses are engaged in B-CLL cells after 2cPE treatment

The microarray studies suggest that a substantial part of genes up-regulated in response to 2cPE are elements of adaptive programs aimed to sustain cell survival. These programs include the accumulation of new proteasomal units, to overcome its obstruction, the up-regulation of chaperones, to counteract misfolding and the potentiation of the anti-oxidant capabilities. In addition, 2cPE triggers also the down-regulation of some signaling pathways. To validate our studies we performed qRT-PCR analysis on four different genes, archetypes of the adaptive responses engaged by 2cPE. Three selected genes were up-regulated: *GCLM*, *HMOX1*, *SQSTM1/p62*, whereas the fourth gene, *LCK*, was down-regulated. Supplementary Table S2 shows the average fold induction and the correlation analysis, respect to the induction of apoptosis in the different B-CLL, for these genes.

*GCLM* (glutamate cysteine ligase modifier) encodes for the component of the glutamate cysteine ligase (GCL), the enzyme responsible for glutathione (GSH) synthesis. Both subunits were increased after 2cPE treatment. The heme oxygenase-1 (*HMOX1*) encodes for an ubiquitous enzyme that supervises cytoprotective responses to toxic insults [24]. *SQSTM1/p62/sequestosome* encodes for a scaffold protein functioning as signaling hub in different pathways including autophagy [25]. Finally, *LCK* encodes for the T-cell-specific member of the Src family of tyrosine kinase, which is also expressed in B-CLL [26]. The qRT-PCR analysis corroborated the regulation of these genes in response to 2cPE treatment (Supplementary Figure S1).

**Figure 3:**
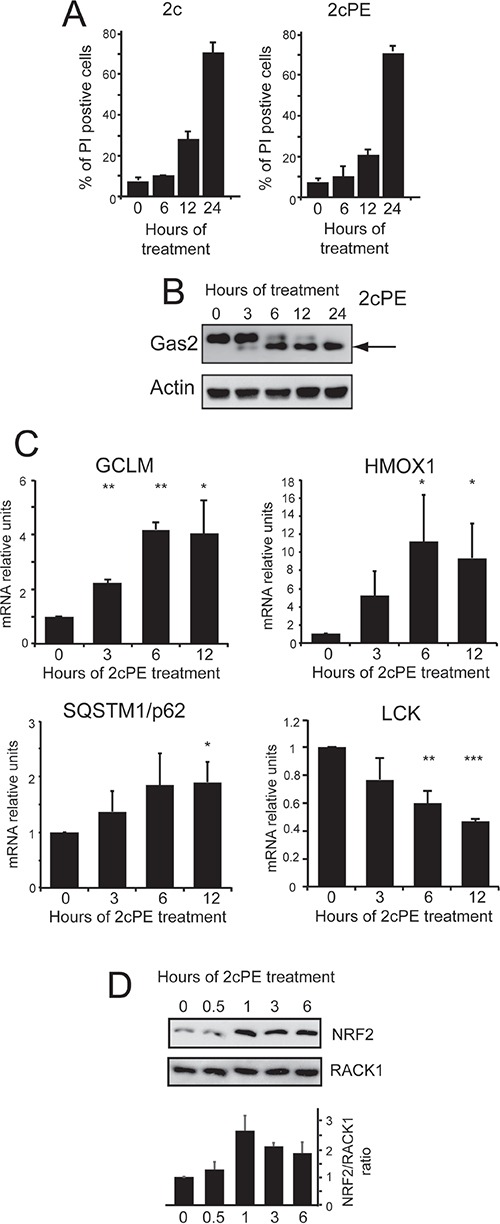
Adaptive responses elicited by 2cPE in MEC-1 cells **A.** Induction of cell death in MEC-1 cells after treatment for the indicated times with 2c (2μM) or 2cPE (4μM). Cell viability was calculated as percentage of cells positive to PI staining using cytofluorimetric analysis. **B.** Caspase-dependent processing of the death substrate Gas2 in MEC-1 cells treated for the indicated times with 4μM of 2cPE. Cellular lysates were generated and subjected to immunoblot analysis. Antibodies anti-Gas2 and anti-actin (as loading control) were used. **C.** Time-course analysis of HOMX, GCLM, SQSTM and LCK mRNA expression levels. MEC-1 cells were treated 4μM of 2cPE for the indicated times and the mRNA levels were monitored by qRT-PCR. All reactions were done in triplicate. **D.** Immunoblot and densitometric analysis of NRF2 levels in MEC-1 cells treated for the indicated times with 4μM of 2cPE. Cellular lysates were generated and subjected to immunoblot analysis using the indicated antibodies. Data were from 2 experiments. Columns, mean loss of viability + SD. *=p<0.05; **=p<0.01; ***p=<0.005.

We next confirmed these data in human chronic B-cell leukemia, MEC-1 cells. The 2cPE pro-drug and its unconjugated version, 2c induce comparable levels of cell death (Figure 3A), thus indicating that this cell line secretes the esterase PLA2G7 [20]. 2c and 2cPE induced cell death was apoptotic, as confirmed by the caspase-dependent proteolytic cleavage of the death substrate GAS2 (Figure 3B) [27]. In MEC-1 cells, similarly to primary B-CLL cells, 2cPE treatment up-regulates the expression of *HMOX1*, *GSLM* as well as of *SQSTM1* and down-regulates *LCK* mRNA levels (Figure 3C).

*HMOX1*, *GCLM* and *SQSTM1* are components of the cytoprotective response to oxidative stress and, likewise other genes induced after 2cPE treatment, they are under the control of the transcription factor NRF2 [28, 29]. Figure 3D proves that 2cPE treatment augmented NRF2 levels in MEC-1 cells.

### Inhibition of the adaptive responses elicited by 2cPE and effects on cell death

**Figure 4:**
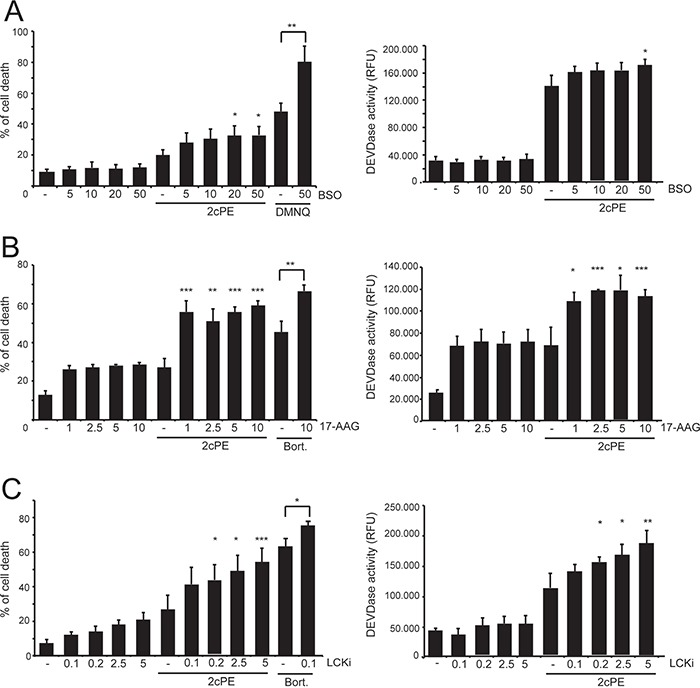
Interfering with the different adaptive responses elicited by 2cPE augmented the apoptotic response **A.** MEC-1 cells were pre-treated with the indicated concentrations (μM) of BSO, for 24 h. Next, 2cPE (1μM), DMNQ (20μM) were added for further 24 h. **B.** MEC-1 cells were pre-treated with the indicated concentrations (μM) of 17-AAG for 1 h. Next, 2cPE (1μM), bortezomib (0.5 μM) were added for further 24 h. **C.** MEC-1 cells were pre-treated with the indicated concentrations (μM) of LCKi for 16 h. Next, 2cPE (1μM), bortezomib (0.5 μM) were added for further 24 h. Cell death was scored by PI staining using cytofluorimetric analysis and caspase activity was measured in parallel. Columns, mean (n = 3); bars, SD. *=p<0.05; **=p<0.01; ***p=<0.005.

Several of 2cPE differentially expressed genes (DEGs) boost adaptive responses devoted for sustaining cell survival. Hence, suppressing these adaptive responses should potentiate apoptosis in response to 2cPE. To verify this hypothesis we co-treated MEC-1 cells with 2cPE in combination with buthionine sulfoximine (BSO), a selective inhibitor of γ-glutamylcysteine synthetase, or with the geldanamycin derivative 17-AAG, an inhibitor of HSP90. BSO alone was unable to trigger cell death but at highest doses it potentiated the pro-death effect of 2cPE, as scored by both PI positivity and caspase activity (Figure 4A). When the ROS generator DMNQ was used, as positive control, the cooperative effect of BSO was more evident (Figure 4A). Treatment with 17-AGG alone induces apoptosis in approximately 30% of cells, independently from the applied dose. When MEC-1 cells pre-incubated with escalating doses of 17-AGG were co-treated with 2cPE, cell death as well as the intensity of caspase activation, were significantly augmented (Figure 4B). Similarly, also bortezomib induced cell death was augmented in the presence of the HSP90 inhibitor (Figure 4B).

Down-regulation of LCK expression might be an important event for the pro-apoptotic activity of 2cPE. Also in this case treatment with an LCK inhibitor (LCKi) might exhibit an additive effect with 2cPE. Figure 3C evidences that co-treatment with 2cPE and LCKi potentiates apoptosis and caspase activation in response to 2cPE.

### Markers of responsiveness to 2cPE treatment

**Figure 5:**
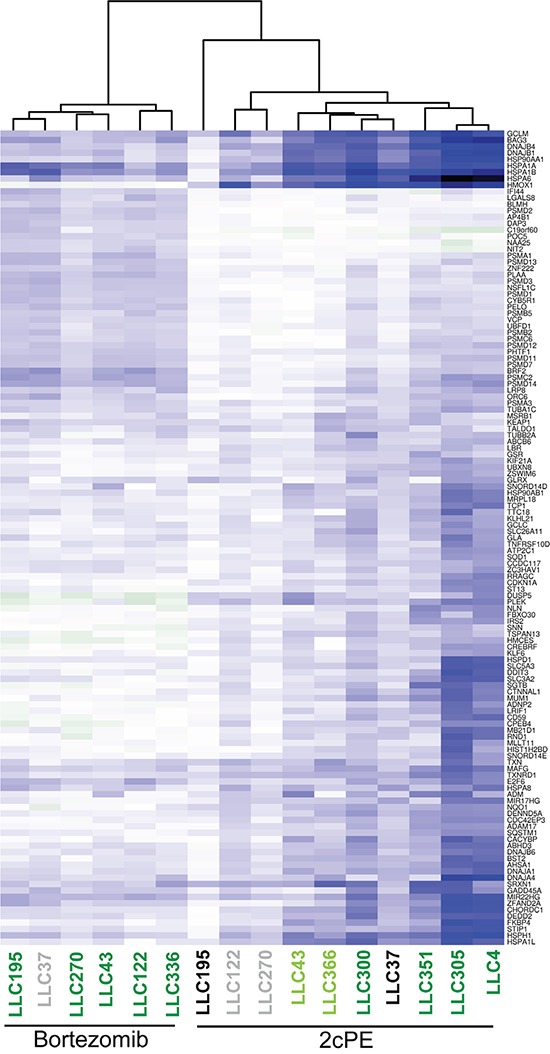
Resistance to 2cPE induced apoptosis correlates with the magnitude of changes in DEGs DEGs induced by 2cPE or bortezomib treatments in B-CLL cells were hierarchically clustered as described in the material and methods. In the heat map up-regulated genes are displayed in blue and down-regulated genes in dark green. A colour code was used to represent the apoptotic responsiveness to 2cPE and bortezomib of the different B-CLL cells. Apoptosis >75% (dark green); apoptosis between 46-74% (light green) apoptosis <20% (grey) apoptosis <10% (black).

The apoptotic responsiveness of B-CLL cells from the different patients to 2cPE is heterogeneous (Table 1). Some cells are highly susceptible and others are resistant. Hence, we decided to use the pattern of all DEGs in all patients to discover markers of 2cPE responsiveness. The lists of DEGs were fused and used to extract the values of single patient differences (fold changes >1.5 <-1.5; *P* values <0.05). An unsupervised clustering analysis was performed and the results are presented as heat map (Figure 5). The apoptotic responsiveness of the different leukemic B-cells is displayed by a colour code. The hierarchical clustering evidences a strong correlation between the magnitude of changes in DEGs and the induction of cell death. In the case of 2cPE treatment, with the exclusion of patient LLC37, all B-CLL cells responsive to 2cPE in terms of apoptosis, cluster together and show elevated fluctuations of DEGs. On the other side, B-CLL cells, which are resistant to 2cPE treatment evidence fewer transcriptional changes and cluster together. The only exception concerns leukemic cells of patient LLC37, which are resistant to 2cPE treatment but cluster together with responsive cells. Interestingly, LLC37 cells are also partially resistant to bortezomib.

### Resistance to 2cPE induced apoptosis in B-CLL cells from patients LLC122, LLC195 and LLC270

The resistance to 2cPE-induced apoptosis, observed in some leukemia B-cells (LLC122, LLC195 and LLC270) is tightly correlated to much lower variations in the transcriptome. This failure could arise from an incapability to process the pro-drug 2cPE in the extracellular environment, as observed in certain cancer cells [20]. mRNA expression levels analysis of PLAG7, the secreted esterase responsible for 2cPE pro-drug maturation, excludes this hypothesis (Supplementary Figure S2).

**Figure 6:**
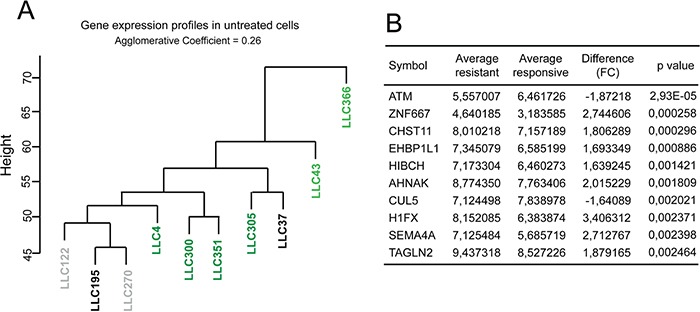
ATM and the resistance to 2cPE **A.** Hierarchical clustering of gene expression profiles of untreated B-CLL cells from the different patients. A colour code was used to represent the apoptotic responsiveness to 2cPE and bortezomib of the different B-CLL cells. Apoptosis >75% (dark green); apoptosis between 46-74% (light green) apoptosis <20% (grey) apoptosis <10% (black). **B.** 10 top DEGs in untreated B-CLL cells from patients LLC122, LLC195 and LLC270 compared to the untreated leukemia cells, responsive to 2cPE, from all other patients. DEGs were ranked for *P* value.

Alternatively, resistance to 2cPE could arise from a common mRNA signature, as generated during disease progression, through the accumulation of peculiar genetic alterations. This signature should predict drug-responsiveness. To verify this possibility, we performed hierarchical gene clustering analysis, of gene expression profiles of untreated B-CLL cells from all patients. Figure 6A shows that, the B-CLL cells of the three patients resistant to 2cPE treatment cluster together. Next, a standard t-test was performed to rank DEGs between 2cPE unresponsive (*n*3) and the responsive leukemia cells (*n*7) (Figure 6B). The *ATM* gene provided the best score (*P* value 2.9306E-005; fold change −1.87). In accordance, all three patients-derived B-CLL cells display the 11q deletion, which affects the *ATM* locus [30]. Other two genes, *NPAT* and *CUL5* lie in the same chromosome region and they also scored a significant reduction in terms of expression in the non-responsive patients (*P* value 0.0035; fold change −1.69 and *P* value 0.0020; fold change −1.64, respectively).

**Figure 7:**
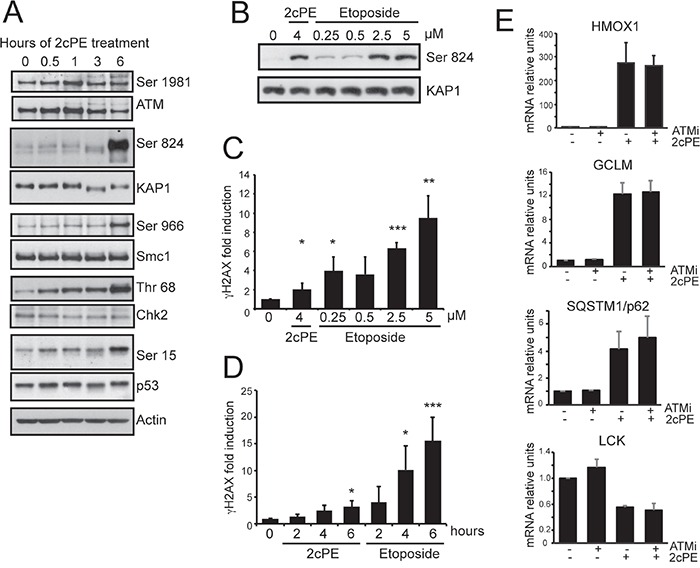
2cPE activates ATM signaling **A.** Activation of the ATM signaling pathway in response to 2cPE treatment. Cellular lysates were generated at the indicated time points and subjected to immunoblot analysis. The detected proteins and their phosphorylated forms are indicated. Actin was used as loading control. **B.** Immunoblot analysis comparing ATM activation in response to etoposide and 2cPE. KAP1 phosphorylation was used as read-out for ATM activation. MEC-1 cells were treated for 6 h with the indicated concentrations of the two drugs. **C.** Quantitative cytofluorimetric analysis of γH2AX positivity as marker of DNA damage induction. MEC-1 cells were treated for 6 hours with the indicated concentrations of the two drugs. **D.** Time-course of γH2AX positivity by quantitative cytofluorimetric analysis. MEC-1 cells were treated for the indicated hours with 4μM of 2cPE or 2.5μM of etoposide. **E.** Time-course analysis of HOMX, GCLM, SQSTM and LCK mRNA expression levels in MEC-1 cells treated with 4μM of 2cPE in the presence of the ATM inhibitor KU-55933. Cells were pre-treated for 1 hour with KU-55933 (10μM), next, 2cPE was added for further 6 h. Columns, mean (n = 3); bars, SD. *=p<0.05; **=p<0.01; ***p=<0.005.

Since ATM is an important signaling molecule, we decided to study whether this kinase is activated in response to 2cPE. Figure 7A demonstrates that the ATM pathway is activated in response to 2cPE, as proved by the phosphorylation of the ATM substrates KAP1, Smc1, Chk2 and p53. The 2cPE-mediated activation of ATM prompted us to evaluate the induction of DNA double-strand breaks. Different concentrations of etoposide were used to induce DSBs and ATM activation. 4μM of 2cPE and 2.5/5 μM of etoposide elicited ATM activation at comparable intensities, as scored by KAP1 phosphorylation (Figure 7B). A robust increase of γH2AX positivity (a marker of DSBs) can be appreciated in cells treated with 2.5 or 5 μM of etoposide. By contrast, only a modest but significant increase in γH2AX positivity was triggered by 2cPE treatment (Figure 7C). We confirmed these data by a time-course analysis. Again a modest, compared to etoposide, but significant increase in γH2AX was detectable in 2cPE treated cells at 6 hour (Figure 7D).

**Figure 8:**
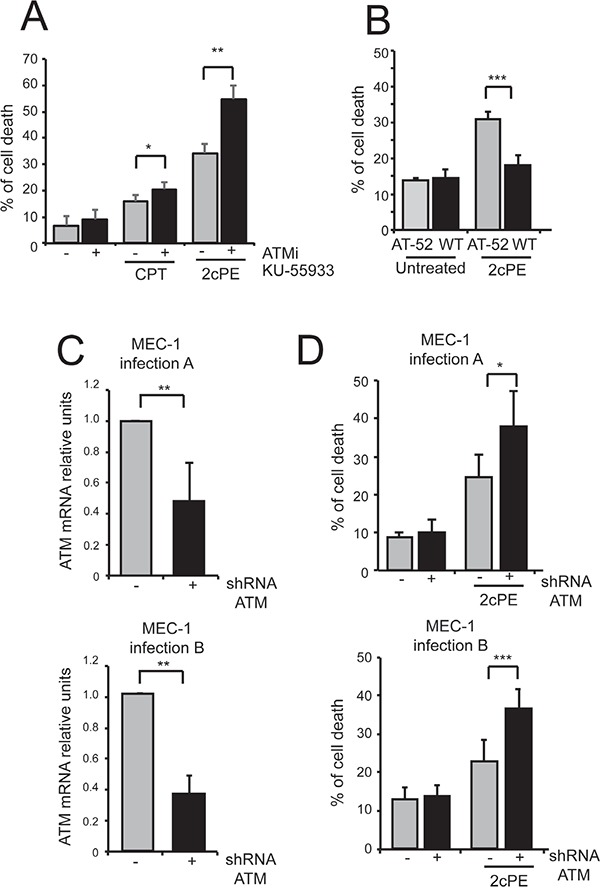
ATM inhibition strengthens apoptosis induced by 2cPE **A.** MEC-1 cells were pre-treated for 1 hour with KU-55933 (10μM). Next, 2cPE (2μM) or camptothecin (CTP) (50μM) were added for further 20 h. Cell death was calculated as percentage of cells positive to PI staining using cytofluorimetric analysis. **B.** LCL cells WT or AT-52 mutated were treated for 22 h with 2μM of 2cPE. Cell death was scored by PI staining using cytofluorimetric analysis. **C.** ATM mRNA levels in MEC-1 cells expressing the shRNA against ATM or the control, as generated by two distinct lentiviral infections. **D.** MEC-1 cells with down-regulated ATM expression generated after two distinct infections (A and B) and control cells were treated with 2cPE (0.5μM) for 6 h. Cell death was calculated as percentage of cells positive to PI staining using cytofluorimetric analysis. Columns, mean (n = 4); bars, SD. *=p<0.05; **=p<0.01; ***p=<0.005.

Next we investigated the contribution of ATM in 2cPE-induced gene expression changes. Inhibition of the ATM kinase using the specific inhibitor KU-55933 did not influenced the 2cPE-mediated up-regulation of *GCLM, HOMX, SQSTM1* and the down-regulation of *LCK* (Figure 7E). Surprisingly, when the effect on cell survival was evaluated, the ATM inhibitor strongly potentiated apoptosis in response to 2cPE (Figure 8A). To confirm this result we used ataxia telangiectasia (AT)-derived lymphoblastoid cell lines (AT-LCLs) WT or lacking ATM protein expression, due to the homozygous mutation AT-52RM [31]. Figure 8B illustrates that cells lacking ATM expression are more sensitive to death induced by 2cPE treatment. Finally, we also generated MEC-1 cells with down-regulated ATM expression after lentiviral infections with a specific shRNA (Figure 8C). MEC-1 cells with down-regulated ATM were more susceptible to apoptosis after 2cPE treatment (Figure 8D).

## DISCUSSION

Although bortezomib has proved inefficacy for the treatment of CLL patients [8], the UPS represents a promising therapeutic target and new UPS inhibitors are under evaluation [32–34]. In this manuscript we have demonstrated that 2cPE triggers apoptosis in B-CLL cells and activates multiple transcriptional programs.

The vast majority of DEGs associated to 2cPE and bortezomib treatments encode for elements of stress response pathways, aimed to sustain cell survival. Interfering with such adaptive pathways potentiates the pro-death effect of 2cPE. This discovery could help to improve the anti-neoplastic efficiency of these molecules, by applying selective co-treatments.

Interestingly 2cPE is a stronger inducer of proteotoxic stress compared to bortezomib. Proteotoxic stress is emerging as an attracting druggable response. Malignant cells exhibit higher levels of proteotoxic stress, as a consequence of their higher proliferative and mutational status [35–38]. This peculiarity renders cancer cells more dependent on the UPS and more vulnerable to its inhibitors or to inducers of proteotoxic stress [39, 40]. We propose that 2cPE and similar compounds are efficient anti-neoplastic drugs *in vivo*, because they elicit a vicious cycle, by triggering proteotoxic stress in cancer cells, which already exhibit high level of such stress. By contrast, 2cPE-induced proteotoxic stress is manageable in normal cells, thus explaining the absence of general toxicity *in vivo* [40–42]. It is possible that 2cPE and similar compounds, in addition of targeting the catalytic cysteine of isopeptidases, react with and consume the glutathione pool, thus provoking oxidative-stress. In fact, NRF2 is activated and the GSH regenerating system is up-regulated in response to 2cPE. Moreover, pre-treatment of G5 with N-acetylcysteine completely abrogates its apoptotic activity (data not shown).

Because of the heterogeneity of this disease [43], some leukemia cells are resistant to 2cPE treatment. Gene expression profile analysis indicates two different conditions of resistance. In the first case, cells display overt changes in DEGs associated to 2cPE (patient LLC37). In the second case, which comprises leukemia cells from patients LLC122, LLC195 and LLC270, DEGs changes are dramatically reduced.

The components of the apoptotic machinery in LLC37 cells, (BCL2 family members and caspases) do not evidence alterations in their expression (data not shown). Hence, currently we cannot explain the origin of such resistance. Importantly, LLC37 cells were analogously resistant to bortezomib.

In leukemia cells from patients LLC122, LLC195 and LLC270, resistance is not due to deficit in pro-drug maturation, as recently observed in other cancer cells [20]. Hierarchical gene clustering analysis of the different patient-derived B-CLL cells and the presence of the 11q deletion suggested that ATM could be a candidate for such resistant phenotype. The ATM kinase has a well-defined role in sensing DNA double-strand breaks (DSB) and transducing downstream signals that activate the DNA repair and the cell cycle checkpoint machinery [44]. Notably, the ATM signaling pathway was stimulated by 2cPE.

ATM is also activated by oxidative stress, through a mechanism distinct to that of activation by DNA breaks [45]. The activation of the anti-oxidants response and the feeble appearance of DSBs in 2cPE treated cells, suggest that a perturbed redox balance might be responsible for ATM activation [46]. In an unexpected manner, ATM inhibition reinforced apoptosis in 2cPE treated cells. Hence, ATM cannot be responsible for the apoptotic resistance to 2cPE. Although we do not have an explanation for such resistance, our results disclose a new opportunity for testing 2cPE in cancer cells that have accumulated mutations in ATM, in p53, or in both genes [47, 48].

In conclusion we have demonstrated that 2cPE triggers pleiotropic cellular stresses. At first glance induction of multiple stresses does not seem to be attractive for the selective elimination of neoplastic cells, however several recent reports about the effectiveness in animal models of this class of compounds [12, 14, 15, 20] justify further studies to improve their anti-neoplastic activities.

## MATERIALS AND METHODS

### Patients, cell separation and culture conditions

The ethic approval for our study was obtained from ethic committee of “Regione Veneto”: (Prot.n. 2662P). We obtained peripheral blood from 10 patients that satisfied standard morphological and immunophenotypic criteria for CLL according to the guidelines of the International Workshop on Chronic Lymphocytic Leukemia IWCLL [49]. No patient was previously treated with bortezomib or other proteasome inhibitors. Peripheral blood mononuclear cells (PBMCs) were isolated as previously described [50]. All samples utilized had a CD19^+^ B-cell content greater than 95%. Purified cells were cultured in RPMI-1640 medium supplemented with 10% heated inactivated fetal calf serum (FCS; Invitrogen, Paisley, UK). Human chronic B-cell leukemia, MEC-1 cells and EBV-immortalized lymphoblastoid cell lines (LCLs) WT and AT52RM and compound heterozygous mutations 7626 C→T/8365 del A, were grown under similar conditions. Mediums were supplied with heated inactivated fetal calf serum (FCS; Sigma Aldrich), penicillin (100 U/mL), glutamine (2 mmol/L), and streptomycin (100 μg/mL).

### Reagents and antibodies

Chemicals used were: bortezomib (LC Laboratories), 2,3-dimethoxy-1,4-naphthoquinone (DMNQ), Propidium Iodide (Sigma Aldrich), buthionine sulfoximine (BSO), 7-cyclopentyl-5-(4-phenoxyphenly)-7H-pyrrolo[2,3-d]pyrimidin-4-amine LCK inhibitor (LCKi), 17-(Allylamino)-17-demethoxygeldanamycin (17-AAG) (Cayman Chemicals), ATM inhibitor (KU-55933) (Abcam Biochemicals), 2c ((*E*)-3,5-Bis[(p-nitrophenyl)methylidene]-4-hydroxycyclohexanone), 2cPE ((*E*)-3,5-Bis[(p-nitrophenyl)methylidene]-4-oxocyclohexyl polyethyleneglycol succinate). Primary antibodies used were: anti-phospho-Ser139 H2A.X, anti-pKAP-1/TIF1β Ser824 and anti-KAP1/TIF1β (Cell Signaling), anti-actin (a2066 Sigma) anti-NRF2 (Santa Cruz Biotechnology).

### RNA expression array and data analysis

Total RNA was isolated using RNeasy Mini kit (Qiagen). RNA samples concentration and purity (assessed as 260/280 nm and 260/230 nm ratios) were evaluated by NanoDrop ND-1000 spectrophotometer (NanoDrop Technologies; Wilmington, DE), while RNA integrity was assessed by using the Agilent 2100 Bioanalyzer (Agilent Technologies; Waldbrunn, Germany). For gene expression profiling (GEP) cDNA synthesis and biotin-labeled target synthesis were performed using the GeneAtlas 3′ IVT Express Kit according to the protocol supplied by Affymetrix. The HG-U219 Array Strips (Affymetrix; Santa Clara, CA) hybridization, staining, and scanning were performed by using the GeneAtlas Platform. All the GEP data have been deposited in the NCBI's Gene Expression (GEO) public repository http://www.ncbi.nlm.nih.gov/geo/query/acc.cgi?token=kbapyckufpcdxwh&acc=GSE74514. Data analysis was performed using the paired t-test as previously described [51]. *P* values were adjusted for multiple testing using the False Discovery Rate method. Differentially expressed genes (DEGs) were selected based on fold change >1.5 and <-1.5 fold and *P* values <0.05. The analysis of Gene Ontology terms was performed using the DAVID server. DEGs in response to 2cPE or bortezomib were clustered using hierarchical clustering by computing the Euclidean distance and applying the complete linkage method. For the hierarchical clustering of the gene expression profiles of B-CLL cells from different patients before drug-treatment the average linkage method was used.

### RNA extraction and qRT-PCR analysis

RNA was extracted using Tri-Reagent (Molecular Research Center) and retrotranscribed by using 100U of Moloney murine leukemia virus reverse transcriptase (Invitrogen). The primer sequences used are available upon request. Quantitative reverse transcription-PCR (qRT-PCR) analyses were performed using Bio-Rad CFX96 and SYBR Green technology. HPRT and GAPDH were used as normalizer genes. All reactions were done in triplicate. Data were from al least 3 experiments ± SD.

### Caspase activation assays and cytofluorimetric analysis

Analysis of caspase-3 activation was performed using Caspase-3 fluorescent assay kit (Cayman chemical). For quantification of cell death, cells were re-suspended in 500 μL of PBS and stained with propidium iodide (PI). Fluorescence was determined with a FACScan™ (Beckman Dickinson). To detect H2AX phosphorylation cells were fixed in paraformaldehyde 3% for 20′ at room temperature, permeabilized with 0.1% TritonX-100 and incubated with the primary antibody anti-pH2AX for 1hr. After washes, cells were incubated with the secondary antibody anti-rabbit Alexa Fluo-488 (Invitrogen) for 30 min. Results were from al least 3 experiments ± SD.

### Western blotting and lentiviral infections

Proteins obtained after an SDS denaturating lysis and sonication were transferred to nitrocellulose membrane and incubated with the specific primary antibodies. Secondary antibodies were peroxidase-conjugated goat anti-rabbit or anti-mouse (Sigma). Blots were developed with Super Signal West Dura (Pierce). For stable ATM down-regulation, the vector-based shRNA interference pLenti6/BLOCK-iT-DEST (Invitrogen) was used as previously described [52]. Culture supernatant obtained from transfected recipient 293 cells, containing viral particles, was recovered and used to infect MEC-1 cells.

## SUPPLEMENTARY FIGURES AND TABLES


