# Supplementary material for: The isopeptidase inhibitor 2cPE triggers proteotoxic stress and ATM activation in chronic lymphocytic leukemia cells
Source: Oncotarget. 2016 May 31;7(29):45429–43. doi: 10.18632/oncotarget.9742 (PMC5216732; doi:10.18632/oncotarget.9742)
Supplement: Supplementary file 1 [file oncotarget-07-45429-s001.pdf]

The isopeptidase inhibitor 2cPE triggers proteotoxic stress and ATM activation in chronic lymphocytic leukemia cells

SUPPLEMENTARY FIGURES AND TABLES

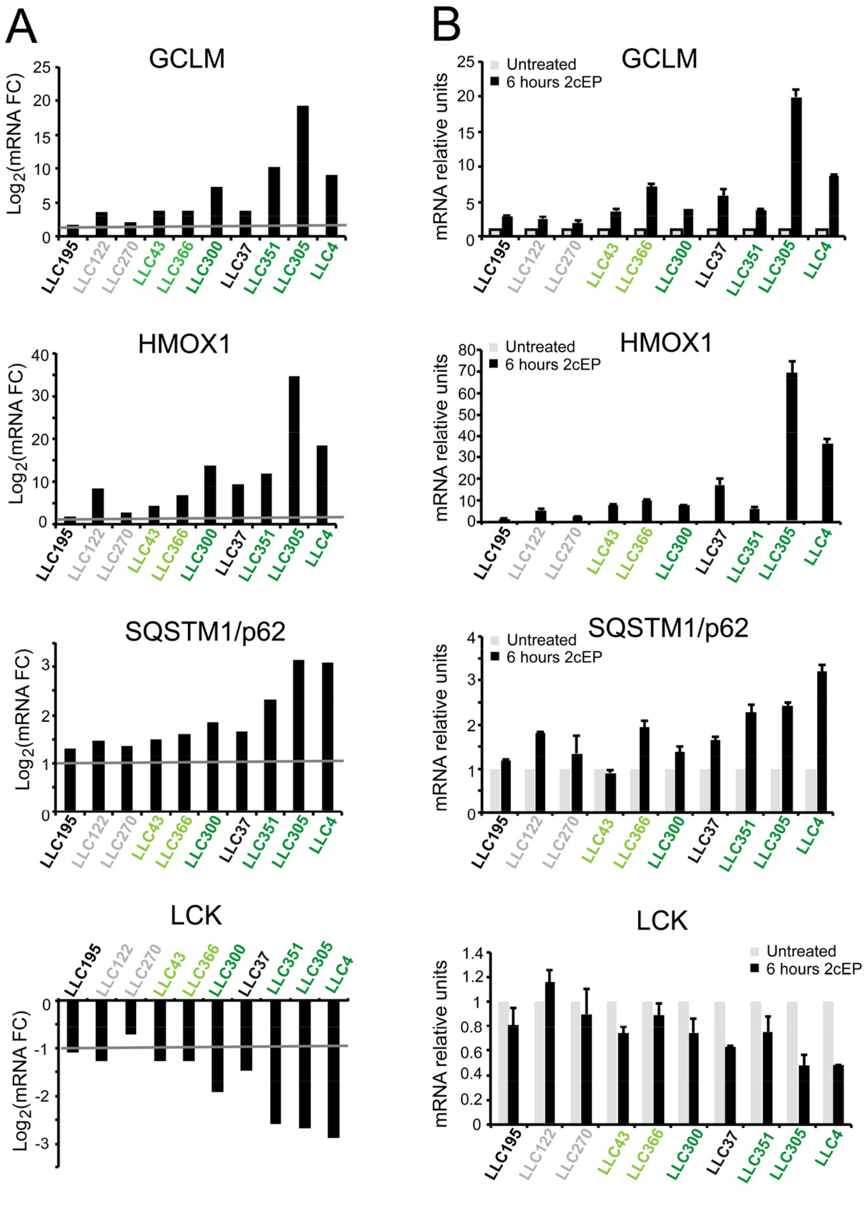

Supplementary Figure S1: Microarray data validation. Analysis of the expression levels of GCLM1, HMOX1, SQSTM1 and LCK in B-CLL from different patients treated for 6 hours with 2cPE. **A.** The original microarray data are represented as Log2 fold changes respect to untreated cells. To represent the apoptotic responsiveness to 2cPE of the different B-CLL, we have used a colour code. Apoptosis >75% (dark green); apoptosis between 46-74% (light green) apoptosis <20% (grey) apoptosis <10% (black). Apoptosis was scored after 24 hours of treatment by cytofluorimetric analysis. **B.** The same mRNAs used for the microarray analysis were tested by qRT-PCR analysis in order to validate the regulation by 2cPE of the different genes.

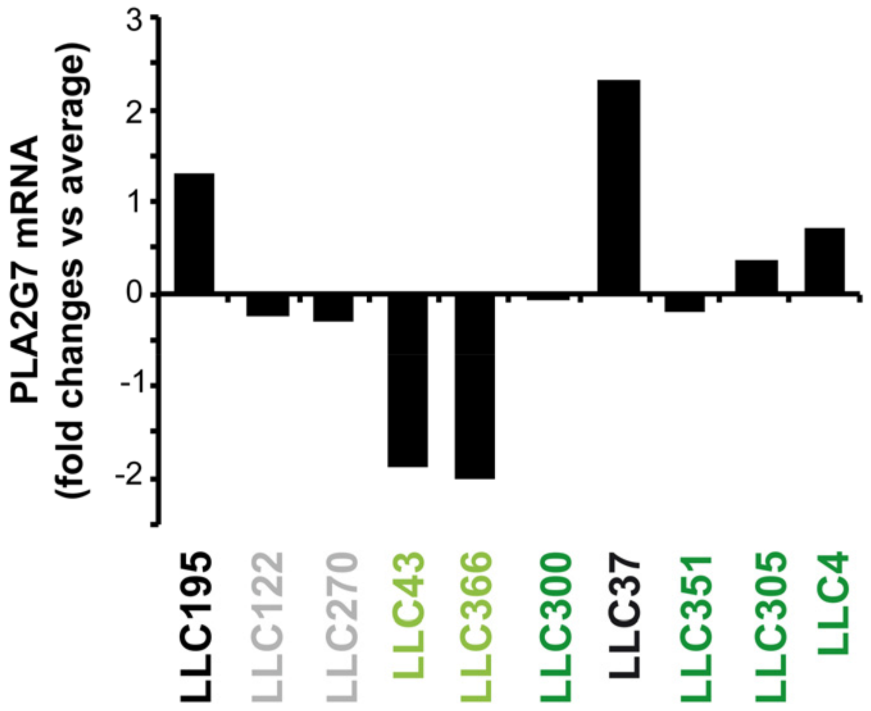

Supplementary Figure S2: PLA2G7 expression in the different B-CLL. PLA2G7 expression levels in B-CLL cells from different patients. Microarray data are represented as Log2 fold changes respect to the average of the relative untreated B-CLL cells. Apoptotic responsiveness to 2cPE was >75% (dark green); between 46-74% (light green) <20% (grey) <10% (black). Apoptosis was scored after 24 hours of treatment by cytofluorimetric analysis.

Supplementary Table S1: Comparative GO enrichment analysis for 2cPE and bortezomib regulated genes in B-CLL cells

Treatment	GO term	count	%	P value	List total	Fold Enrich.	Bonferonni	Benjamini	FDR
2cPE	GO:0006986~response to unfolded protein	13	8,333333333	1,66013E-12	125	19,81566197	2,67113E-09	2,67113E-09	2,78174E-09
Bortezomib	GO:0006986~response to unfolded protein	7	7,865168539	2,17473E-06	74	18,02360107	0,001868522	5,50064E-05	0,003369876
2cPE	GO:0010033~response to organic substance	25	16,02564103	3,1083E-08	125	3,752565881	5,00113E-05	1,25031E-05	5,20837E-05
Bortezomib	GO:0010033~response to organic substance	10	11,23595506	0,015138055	74	2,535517487	0,999997992	0,226802704	21,05119819
2cPE	GO:0006457~protein folding	13	8,333333333	7,7335E-08	125	7,948655367	0,000124424	1,77759E-05	0,000129585
Bortezomib	GO:0006457~protein folding	5	5,617977528	0,015312227	74	5,164147198	0,999998275	0,225238107	21,26727373
2cPE	GO:0042981~regulation of apoptosis	25	16,02564103	2,38466E-07	125	3,365174129	0,000383618	4,79604E-05	0,000399581
Bortezomib	GO:0042981~regulation of apoptosis	12	13,48314607	0,003695497	74	2,728519564	0,958580954	0,071371811	5,575624266
2cPE	GO:0043066~negative regulation of apoptosis	16	10,25641026	8,25416E-07	125	4,891480226	0,001327213	0,000110668	0,001383084
Bortezomib	GO:0043066~negative regulation of apoptosis	8	8,988764045	0,002933021	74	4,131317758	0,920030058	0,061199809	4,449609262
2cPE	GO:0000502~proteasome complex	4	2,564102564	0,014808584	109	7,68957738	0,942988966	0,436113387	16,95169676
Bortezomib	GO:0000502~proteasome complex	14	15,73033708	4,31329E-18	69	42,51556189	5,26221E-16	5,26221E-16	4,96617E-15
2cPE	GO:0051340~regulation of ligase activity	5	3,205128205	0,006499263	125	6,680493827	0,999972227	0,116115393	10,35019702
Bortezomib	GO:0051340~regulation of ligase activity	15	16,85393258	6,41469E-18	74	33,85385385	5,51663E-15	2,75832E-15	9,94011E-15
2cPE	GO:0010498~proteasomal protein catabolic process	4	2,564102564	0,067194175	125	4,244078431	1	0,450363568	68,82457044
Bortezomib	GO:0010498~proteasomal protein catabolic process	15	16,85393258	1,90722E-16	74	26,88394277	1,90958E-13	1,90958E-14	3,44169E-13
2cPE	GO:0043085~positive regulation of catalytic activity	13	8,333333333	0,002929051	125	2,7056	0,991082671	0,066115305	4,796372764
Bortezomib	GO:0043085~positive regulation of catalytic activity	19	21,34831461	1,93518E-10	74	6,67962578	1,66426E-07	8,75925E-09	2,99873E-07
2cPE	GO:0007049~cell cycle	15	9,615384615	0,011551262	125	2,091958763	0,999999992	0,170507825	17,69046796
Bortezomib	GO:0007049~cell cycle	17	19,1011236	2,85459E-06	74	4,00487601	0,002451938	7,01389E-05	0,004423333

Supplementary Table S2: Fold induction (Log2) and correlation analysis of HMOX1, GCLM, SQSTM1 and LCK genes in response to 2cPE treatment in B-CLL cells

Gene	Fold induction*	P value	Correlation r°	P value
HMOX1	7,901522999	0,014238567	0.77667	0.220759
GCLM	5,000813182	0,014238567	0.75	0.240501
SQSTM1	1,750845312	0,018787535	0.81667	0.192995
LCK	-1,637596008	0,027882895	-0.78333	0.213217

*Average fold induction, °Correlation between the fold changes and the induction of apoptosis.
